# Chloral Inebriety

**Published:** 1880-03

**Authors:** J. B. Mattison


### ARTICLE VI.

### *Chloral Inebriety.*

BY DR. J. B. MATTISON.

Inebriety from chloral, like that of opium and alcohol, proceeds from one of two causes—its professional employment till the morbid demand for constant use is created, or else its self-administration till the same result is reached. I have expressed my belief that the share of professional responsibility in the production of opium inebriety is very great, and have no reason to change that opinion. With alcohol and chloral I think it less. Chloral seems to possess a special adaptability for self-taking; it has such a wonderful power in bringing sleep and mental worry and jar, the reverse of which is so often prominent in almost every department of active life.

Again, I believe the laity look upon chloral with less distrust and as less dangerous than opium; and therefore are more disposed, when in pain or sleepless, to act as their own medical advisers, which, in the present lax restrictions regarding the sale of it and other narcotics, can be accomplished with little or no difficulty.

Then, too, chloral is more efficient than opium in overcoming the tremors, wakefulness, and general dilapidation following a drunken pout, and as such may be used to an extent establishing an additional form of inebriety, although there is a ludicrous lack of truth in the assertion of a Dutch journal, some time ago, "that a large proportion of the so-called 'drinks' which are used in America contain chloral hydrate."

Dr. Richardson asserts that six months after the introduction of chloral in England, its use, outside of the profession, had become quite extensive, and in 1871 he felt impelled to make a report on the subject before the British Association for the Advancement of Science.

Dr. Wm. B. Atkinson states "that when in California, in 1871, he found it in almost universal use. Persons with no pretence to any medical knowledge were constantly employing it in large doses for the relief of neuralgic pains." Dr. Squibb, however, express his belief that the non-professional use of it has largely diminished of late years. Doubtless its not infrequently fatal effects have had much to do with the decrease; and it will prove fortunate if, even at such expense, its use be relegated to professional hands, where it properly belongs.

One reputed quality of opium and alcohol, which is sometimes a pretext for indulgence and a factor in their respective forms of inebriety, does not pertain to chloral—

that is, the power to stimulate mental activity. It dulls and depresses, or, if otherwise, the latter is early and transient.

The prognosis of chloral inebriety is variable. Dr. T. D. Crothers asserts it unfavorable. The disease is less frequent than opium, and much more infrequent than alcoholic inebriety, and I am not aware of any data as to the proportion of recoveries. The danger is a more or less speedy relapse. Much will depend on the length and degree of addiction, freedom from painful organic complication, absence of alcohol or opium taking, individual constitution, and surroundings subsequent to cure.

Another point to be noted is that chloral habitués are exposed to a special danger. While the habitual use of opium admits of its gradual increase without risk, so that enormous doses can be taken with impunity, that of chloral is sometimes the reverse, and serious effects have followed the use of a smaller dose than the patient had for some time been accustomed to taking.

It must not be supposed that all habitual chloral-takers are inebriates, strictly speaking. The subject, like that of alcohol and opium using, has a double aspect—a vice and a disease. A person who habitually indulges in either of these agents, and yet retains sufficient will-power to abandon them, is simply vicious—a proper object of moral suasion; but when volition ends, disease—inebriety—begins.

*Treatment.*—Either of two methods will succeed in the treatment of this affection—entire and immediate chloral withdrawal, or its more gradual abandonment. The same objection to the former plan presents as in opium inebriety; it causes a profound shock to the system, intense reflex irritation, and great distress of mind and body. By the latter method the same result is reached with much less suffering, and I regard it wiser and more humane.

If the heroic plan be adopted, the nervous disturbance may be expected to subside in from four to seven days. To lessen it, hypodermic morphia, Indian hemp, hyoscyamus, donium, the bromides, hot baths, and other nervines may be employed. It is important to remember that after habitual indulgence in opium or chloral, much larger doses than usual of other sedatives are essential to produce any effect. The nervous system seems to acquire a special tolerance that renders ordinary doses absolutely inert.

With the sedative treatment must be used a tonic course, especially strychnine, as a nerve strengthener, and iron, preferably the muriated tincture, to enrich the impoverished blood. The observations of Keyes, Thompson, and others would seem to favor the use of hydrarg. bichlor, as an additional agent in restoring the red corpuscles. Full nourishing diet, electricity, out-of-door exercise, and cheerful social surroundings are essential roborative auxiliaries. —*Druggists Circular.*

